# Prevalence of use of on-label and off-label psychotropics in the Greek pediatric population

**DOI:** 10.3389/fphar.2024.1348887

**Published:** 2024-03-14

**Authors:** Stella Pesiou, Rafel Barcelo, Georgios Papazisis, Ferran Torres, Caridad Pontes

**Affiliations:** ^1^ Departament of Pharmacology and Toxicology, Universitat Autònoma de Barcelona, Barcelona, Spain; ^2^ European Medicines Agency, Committees and Quality Assurance Department, Human Medicines Division, Amsterdam, Netherlands; ^3^ Biostatistics Unit, Medical School, Universitat Autònoma de Barcelona, Barcelona, Spain; ^4^ Clinical Research Unit and Department of Clinical Pharmacology, School of Medicine, Aristotle University of Thessaloniki, Thessaloniki, Greece; ^5^ Digitalization for the Sustainability of the Healthcare System DS3–IDIBELL, Barcelona, Spain; ^6^ Clinical Pharmacology Service, Hospital de la Santa Creu i Sant Pau, Barcelona, Spain

**Keywords:** children, adolescents, pediatric, psychiatry, psychotropics, off-label, Greece

## Abstract

With a global increased use of psychotropics in pediatrics, their off-label use is a concern due to uncertainty and risk. Data on psychotropics use in the Greek pediatric population do not exist to date. We analyzed retrospective data from the nationwide pharmacy claim database, to estimate the prevalence of psychotropics in pediatrics focusing on off-label use (March 2016-October 2019). In total 63,782 pediatric subjects had at least one identified psychotropic prescription. The prevalence of psychotropics use was 5.1–14.6/1,000 pediatric inhabitants. When excluding 42,508 subjects who received only short-time hydroxyzine, the prevalence was 3.1–6.5/1,000 pediatric inhabitants; adolescents and boys had higher exposures. An in-depth analysis of 21,274 subjects accounting for 222,307 psychotropic dispensations, showed antiepileptics as the most frequent psychotropics, consistently with the finding on epilepsy being the most frequent diagnosis; diazepam was the single drug with the highest exposure in almost all ages. 14% of subjects have received at least one medicine with no pediatric information in the labelling, corresponding to 5.5% of dispensed psychotropics. 7.6% of all dispensed psychotropics were used in a non-authorized age range with quetiapine being the most frequent psychotropic as off-label age range; antipsychotics and antidepressants were first as off-label for non-authorized indication. Data from Greece show that a relevant number of patients are prescribed psychotropics, with 1 in 7 being under off-label use. Due to the limitations inherent to pharmacy claims databases, further research using clinical data for a longer follow-up period could confirm and identify trends in psychotropics use in Greece.

## 1 Introduction

Identified unmet medical needs observed in pediatric subspecialty care, such as pediatric psychiatry, can affect adulthood since healthcare influences the physical and emotional development of children and subsequently their capacity to reach a full potential as adults (National Research Council US Institute of Medicine US et al., 2009; Signorini et al., 2017). By the burden of mental health disorders concerning the pediatric population it is estimated that around 20% of children and adolescents suffer from a mental health condition, having suicide as the third leading cause of death in older adolescents (World Health Organization, 2013; World Health Organization, 2023).

For long periods, non-pharmacological and pharmacological treatments aiming to treat mental conditions in the pediatric population did not receive great attention. With almost no dedicated pharmacological research, the evidence available to inform treatment decisions for children diagnosed with a mental condition has been very limited, and based on clinical experience gained by using medicinal products out of the authorized indications, dosages or patient populations, that is, off-label (Aronson and Ferner, 2017). To overcome the reluctance to conduct research in children, regulatory initiatives have helped to advance the available treatment options, and progressively the medical and scientific communities have realized that it is not ethically acceptable to have lower standards in drug therapy for children and adolescents as opposed to adults. Meanwhile, a large list of reasons has been acting as a significant barrier regarding pharmacological treatments to obtain evidence-based treatment options for children (Koelch et al., 2008; Hoffman et al., 2014), rendering off-label use very common. Quantifying off-label use is difficult, since real-world data concerning the use of medicines for the treatment of mental health conditions in pediatrics in Europe is in general fragmented (World Health Organization, 2013). Studies have reported very different estimates for prevalence of off-label drug use in hospitalised children, from 2% to more than 95% (Balan et al., 2018). For outpatient children, differences in the national health systems, difficulties in differentiating diagnosis in this vulnerable population and social stigma (World Health Organization, 2013) add difficulties to the task of estimating the magnitude and characteristics of outpatient pediatric exposure to off-label medications.

According to recent data, the state of a long financial crisis along with a refugee crisis in Greece seem to have increased the psychological stress in children living in the region compared to the rest of Europe (UNICEF Greece, 2020). However, broad and exact data on epidemiology of mental disorders in the Greek pediatric population do not exist, and no data on relevant treatments and their adequate use are available. Therefore, in this study we investigated the use of psychotropics in children and adolescents on a national level aiming to explore for the first time the exposure of pediatric population to psychotropic medicines and their use as per the authorised conditions by using data from the Greek nationwide prescription database.

## 2 Materials and methods

This was a retrospective observational population-based study of psychotropic consumption in the population under the age of 18 years residing in Greece for the period between March 2016 and October 2019 using anonymized pharmacy claims data on psychotropics from the nationwide electronic prescription database. This database is managed by the Greek e-Government Center for Social Security Services (IDIKA S.A.) which covers almost the entire Greek population (97%–98%, except people without a social security number) (ΗΔΙΚΛ, 2012; Yfantopoulos and Chantzaras, 2018; Bakirtzis et al., 2020). The dataset contains demographics, the unique citizens’ social security number, information on the prescribed medicines (classified using the Anatomical Therapeutic Chemical Classification, ATC), the relevant diagnosis (classified with International Classification of Diseases 10th Revision, ICD-10) connected to the dispensed medicine, as well as the prescribing physician and the geographical region. We did not obtain any information on the prescribed dose and dosing schedule, apart from the number of prescribed boxes. Under psychotropics we considered the defined by WHO ATC groups of antiepileptics (N03A), antipsychotics (N05A), anxiolytics (N05B), hypnotics/sedatives (N05C), antidepressants (N06A), psychostimulants (N06B), psycholeptics and psychoanaleptics in combination (N06C), and drugs used in addictive disorders (N07B).

We reviewed the quality of the received data, and no duplicate cases were detected. We described the psychotropic consumption as annual prevalence defined per 1,000 Greek inhabitants aged below 18 years with at least one psychotropic dispensed by the pharmacy following a prescription during the study period. Prevalences were estimated using the population data from the Hellenic Statistical Authority as the denominator (Hellenic statistical authority, 2021a). We also estimated the annual prevalence stratified by age group and sex.

As regards the off-label use, this was quantified as dispensed psychotropics outside of the authorised age range (if any), based on the information retrieved from the medicinal products labelling and described using percentages. Off-label dispensations for the year 2017 were analyzed in depth. Dispensation:patient ratios (D/P) were calculated to quantitate repeated use by patient. A qualitative sub-analysis on the off-label use of psychotropics checked if the diagnosis in prescriptions were matching or not with the approved indications as reflected in the product labeling. The sub-analysis focused on the most frequently dispensed medicines and the most frequent and relevant single ICD-10 codes in prescriptions.

The statistical package SAS v9.4 (SAS Institute, Cary, NC, United States of America) was used to perform the statistical analysis.

According to the national legislation, the protocol was approved by the relevant ethics committee in Greece, and waiver for consent was granted since this was an observational study using anonymous data; there was no requirement to obtain specific authorisation from the respective national authority.

## 3 Results

### 3.1 Prevalence of psychotropics use and off-label use

For the almost 4-year period, our study population consisted of 63,782 pediatric subjects with at least one dispensed psychotropic covered by the Greek health reimbursement system. Data were available only since March in the year of deployment of the information system (2017), with prevalence estimates of psychotropic use of 5.1 per 1,000 pediatric patients; the following years the annual prevalence ranged between 13.4 and 14.6 per 1,000 pediatric inhabitants in 2019 and 2018 respectively.

The first analysis revealed that there was a high number of study subjects receiving only hydroxyzine, an active substance that belongs to the group of anxiolytics but is mostly used as antihistamine/antiallergic treatment. We excluded the pediatric subjects with hydroxyzine-only dispensations to avoid biases, so that the target group was more homogeneous and representative concerning the use of psychotropics for predominantly mental health disorders. Excluding hydroxyzine, the resulting target population consisted of 21,274 pediatric subjects with at least one dispensed psychotropic. The prevalence of use ranged between 3.1 and 6.5 per 1,000 pediatric inhabitants in 2016, and both 2018 and 2019 respectively. Boys were more exposed than girls to psychotropics throughout the almost 4-year period. Both young and late aadolescents had higher chance to receive a psychotropic than younger age ranges. Details on the prevalence of use in Greece is shown in Table 1.

**TABLE 1:** Prevalence (per 1,000 pediatric inhabitants) of psychotropics use in the Greek pediatric population from 2016 to 2019.

	2016^a^	2017	2018	2019^+^
Number of pediatric inhabitants	1,876,718	1,878,388	1,872,031	1,861,740
Any exposure (prevalence per 1,000 pediatric inhabitants)				
Total	5.1	13.9	14.6	13.4
Girls - Boys	4.5–5.5	12.8–15.0	13.5–15.6	12.4–14.4
Target exposure (prevalence per 1,000 pediatric inhabitants)				
Total	3.1	6.2	6.5	6.5
Girls - Boys	2.7–3.5	5.5–6.8	5.7–7.3	5.7–7.2
< 1 year	0.0	0.9	1.1	1.0
Girls - Boys	0.0–0.0	0.8–1.0	1.1–1.2	0.9–1.0
1–2 years	1.4	6.0	5.6	5.6
Girls - Boys	1.4–1.3	5.8–6.1	5.1–6.0	5.1–6.1
3–5 years	1.5	4.8	5.4	5.7
Girls - Boys	1.3–1.7	4.3–5.3	4.8–6.0	5.0–6.3
6–8 years	2.3	4.5	4.7	4.5
Girls - Boys	2.0–2.6	3.9–5.1	3.9–5.5	3.7–5.2
9–11 years	3.7	6.1	6.1	6.3
Girls - Boys	3.0–4.3	4.9–7.2	5.0–7.2	5.0–7.6
12–14 years	4.4	7.1	7.5	7.2
Girls - Boys	3.5–5.1	5.7–8.5	6.1–8.8	6.0–8.3
15–17 years	5.7	9.9	10.7	10.6
Girls - Boys	5.2–6.3	9.6–10.1	10.3–11.1	10.6–10.6

a : from March to December 2016, +: from January to end of October 2019. Prevalence expressed per 1,000 inhabitants.

**FIGURE 1:**
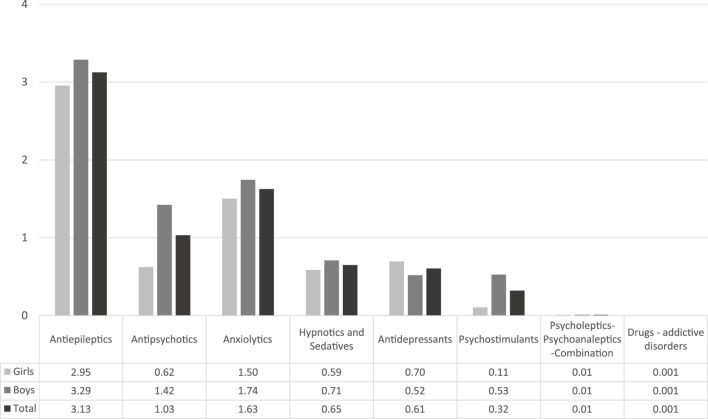
Prevalence (per 1,000 pediatric inhabitants) of psychotropics use by ATC groups.

The ATC group of antiepileptics (N03A) had the highest prevalence rate, followed by anxiolytics (N05B) and by antipsychotics (N05A). Figure 1 below presents the prevalence rates per ATC group for Greece for the corresponding full study period. The most frequent drugs used in our study population were diazepam, followed by two antiepileptics (levetiracetam and valproic acid); some antipsychotics were among the most frequent used medicines (see Supplementary Material).

**FIGURE 2:**
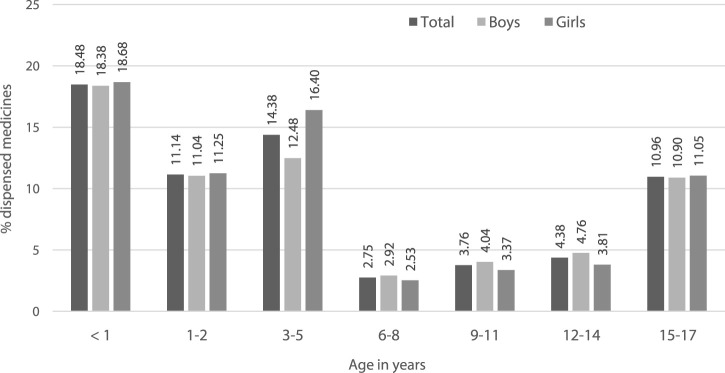
Off-label use as per the labelled age-range, analysis by age strata of pediatric subjects.

Sixteen dispensations corresponding to 5 subjects were excluded from the off-label sub-analysis as the exact ATC code at level 7 could not be identified and thus the off-label status would not be possible to define. Hence, the Greek study subpopulation for this analysis was of 21,269 pediatric subjects with at least one psychotropic dispensed from the Greek health reimbursement system corresponding to 223,307 medicine dispensations. 14% of pediatric subjects used at least one psychotropic as off-label with girls been more exposed to a psychotropic under unauthorised conditions (Table 2). The off-label use was higher in younger populations as described in Figure 2.

**TABLE 2:** Off-label dispensations considering labelling age range and most frequently off-label drugs.

a. Number of patients exposed considering labelling age							
		Girls	Boys			Total	
		n (%)	n (%)			n (%)	
In the authorised age range		7,782 (83.17)	10,501 (88.15)			18,283 (85.96)	
Out of authorised age range		1,575 (16.83)	1,411 (11.85)			2,986 (14.04)	
Total number of patients		9,357	11,912			21,269	
b. Most frequent off-label psychotropic drugs dispensed in 2017							
ATC				Dispensations (n = 119,976)			
Code	Name			n	%		D/P^a^
N05AH04	quetiapine			1,826	1.52		5.7
N03AF02	oxcarbazepine			832	0.69		6.2
N05CH01	melatonin			748	0.62		7.9
N05BA12	alprazolam			677	0.56		2.7
N05AX12	aripiprazole			538	0.45		4.2
N06AB10	escitalopram			514	0.43		3.7
N05AH03	olanzapine			477	0.40		6.0
N05BA09	clobazam			350	0.29		5.1
N06AB04	citalopram			341	0.28		3.4
N03AX16	pregabalin			312	0.26		3.7

^a^ D/P = number of dispensations per patient.

Data concerning the off-label dispensations were analyzed in depth for the year 2017, showing that quetiapine was the single drug with the highest off-label use, while the rest of the medicines were below one per cent of off-label use. Oxcarbazepine ranked second, and melatonin was in the third place; melatonin had a D/P reaching up to almost eight off-label dispensations per patient, making it the highest D/P among all medicines. Olanzapine was the second product in terms of D/P, with six off-label dispensations per patient, but an overall prevalence of off-label use being below 1%. More details can be found in Table 2.

In our sample, three active substances, i.e., levetiracetam, valproic acid and diazepam, were often dispensed as off-label as per indication since prescribers did not specify a diagnosis connected to these dispensations. Valproic acid and aripiprazole were dispensed for pervasive development disorder (PDD) for which they have no authorization. Risperidone, an active substance authorized in Europe for the short treatment of persistent aggression in conduct disorder from the age of 5 years and adolescents with subaverage intellectual functioning or intellectual disability, was the active substance with the highest number of off-label indications, including PDDs, attention deficit hyperactivity disorder (ADHD), obsessive compulsive disorder (OCD) and unspecified psychosis not due to a substance or known physiological condition. Fluoxetine is authorized in major depressive episodes for children and in OCD for adults, but it was used frequently outside of the authorized age range in our sample for depression, and also used in children with anxiety, an indication for which fluoxetine does not have an authorization in Europe. Sertraline is authorized in pediatric patients with OCD while in adults is also authorized for depression, panic disorder (with or without agoraphobia), social anxiety disorder and post-traumatic stress disorder (PTSD); in our sample, sertraline was used in off-label indications for pediatric subjects such as anxiety or depression. Other relevant off-label uses as per indication included alprazolam used in children suffering from anxiety, escitalopram used in children with depression and quetiapine used in children with psychosis, all of which do not have a pediatric indication.

## 4 Discussion

### 4.1 Use of psychotropics in children and adolescents

Medicines are amongst the different therapeutic strategies available to treat mental health disorders. However, for decades medicines have been barely studied in children, making their use for long time mostly empirical based on extrapolation from adults, using formulations not suitable for children, and prescribing them out of the authorized labelling conditions, namely, the off-label use (Commission to the European Parliament and the Council, 2017). Information on the safe and effective use of medicines in this vulnerable patient population is frequently missing, and real world evidence may be able to fill in some of the gaps, e.g., by informing on and extent of off-label use.

Epidemiologic data from Greece are scarce if not null. In the current study using anonymous pharmacy claims data reimbursed by the national health system we report the prevalence of psychotropics use in the Greek pediatric population. Roughly 1.4% of pediatric inhabitants were found to be exposed to psychotropics, but this was mainly due to a use of short-term hydroxyzine, an H1 antihistaminic which is mainly used to treat acute skin allergy indications. By removing the hydroxyzine-only dispensations, the prevalence of pediatric use was below 1%, with adolescents and boys more exposed to psychotropics than younger groups and girls, respectively. Besides adolescents, the youngest age groups (1–5 years old) had higher exposures than older groups (6–8 years old). Antiepileptics were found to be the most frequently used ATC group while diazepam was the most prevalent active substance.

The psychotropic exposure rates we describe in the Greek pediatric population are amongst the lowest estimates considering other regions both in and outside Europe (Zito et al., 2003; Safer et al., 2004; Zito et al., 2008; Zoëga et al., 2009; Kovess Masfety et al., 2015; Pesiou et al., 2023); no previous Greek data were identified in the literature to check for consistency or to compare any change in the trends. In particular, psychostimulants use was the lowest among other regions (Schirm et al., 2001; Faber et al., 2005; Vinker et al., 2006; Zito et al., 2008; Pottegård et al., 2012; Bachmann et al., 2017; Pesiou et al., 2023), approaching the rates reported in Italy (0.1–1.9 per 1,000) (Piovani et al., 2016). It is possible that our data source underestimated exposure bias, but the database collects information from a model for reimbursement that is almost universal, with coverage over 97.3% of the Greek population (ΗΔΙΚΛ, 2012; Yfantopoulos and Chantzaras, 2018; Bakirtzis et al., 2020). Lower prevalence of use in Greece may also be related to differences in the health systems and reimbursement models, as well as geographical and societal factors. Greece is considered among the European countries with the lowest depression rates and the one with the lowest suicide rate, although whether this may reflect certain degree of underdiagnose and stigma cannot be assessed (Eurostat, 2018).

Of note, and distinctively to other databases, the Greek prescription information includes the treated indication, allowing for a qualitative analysis of the reason for drug use. However, relevant data on the diagnostic rates of mental disorders in the pediatric population are not available through the national system. The available information is mostly derived from surveys that are not covering the whole pediatric population (Anagnostopoulos and Soumaki, 2013; Paleologou et al., 2018; Hellenic Statistical Authority, 2021b). In this way we cannot know how many diagnosed patients in need of medication exist in Greece, nor whether the current situation reflects appropriate use, underuse or overuse of psychotropics. In addition, access to mental care may be limited in certain areas mostly due to financial and geographical reasons: attention of patients by mental health specialists is easier in big urban areas but harder otherwise, with a need to move between regions to access specialists; thus, patients may remain undiagnosed and subsequently untreated (Souliotis et al., 2017). Another problem identified in the Greek community is the high stigma on mental health diseases (Tzouvara et al., 2016). The stigma may be even higher when it comes to the pediatric population, and in combination with the difficulties related to the financial crisis in the country, may result in a lot of children undiagnosed and untreated. Even in cases where the mental illness is diagnosed, it is possible that some psychotropic medicines are prescribed under the name of the parents or bought directly in the pharmacy. It is a known fact among the Greek population that direct purchase of prescription-only medicines occurs in the country, except from the ones controlled/restricted under the law for narcotics (i.e., benzodiazepines, opioids). This may be attributed to the economic burden that the costs of medical visits to obtain prescriptions or refills represent to a part of the population, and the fact that the cost of the non-reimbursed drug is lower than that of a medical visit. Therefore, the number of psychotropics dispensed through the national reimbursement system may underestimate the actual consumed amount.

Consumption of antidepressants and hypnotics/sedatives in the general population of Greece was found to be lower than the average of European countries (Institute for Health Economics i-hecon, 2020) which can also confirm the observed high gap in the prevalence rates between Greece and other countries. We included in the study antiepileptic drugs because they are chronic medicines that may also impact neuropsychiatric development, and sometimes are also used to treat some mental health diseases. Epilepsy, however, was their main use in our sample. Greek citizens seem to be more familiar with epilepsy (Diamantopoulos et al., 2006), a neurological disorder whose symptoms are visible and easily spotted as compared to mental disorders, therefore it can be diagnosed early, the need for specialist supervision is unquestioned, and the stigma from the community seems low (Diamantopoulos et al., 2006), resulting in treating options to be broadly acceptable by the parents. In this way, dispensed antiepileptics have less risk to remain underestimated. Compared to previously reported rates of antiepileptics’ prevalence, Greece had similar ones to those reported in the Netherlands (3.7-4 per 1,000) (van de Vrie-Hoekstra et al., 2008; Zito et al., 2008) and Germany (3.8 per 1,000) (Zito et al., 2008), while in Spain (4.97 per 1,000) (Pesiou et al., 2023) and the United Kingdom (7.3–8.69 per 1,000) the reported rates were higher. (Ackers et al., 2007). Comparing to reported prevalence in the United States of America, there is a wide range in the reported rates (1.1–20 per 1,000) depending on the dataset (region, age range, source of data and year), but our rates were slightly within these limits (Zito et al., 2003; Zito et al., 2006; Zito et al., 2008).

The off-label use of psychotropics in the pediatric populations has been previously reported in several countries. Our study also confirmed that a percentage of the dispensed psychotropic medicines was used outside the authorized conditions. In the analyzed subset corresponding to data in 2017, around 1 in 7 (14%) of children exposed to psychotropic drugs in Greece had received at least one medicine that had no pediatric information in the product labelling. The percentage of the overall off-label dispensations based on lack of pediatric recommendation in the SmPC was 5.5% in Greece; if the range of the recommended age was taken into consideration, then the percentage increased into 7.5%, with the younger populations mostly exposed to off-label dispensations as well as girls. Comparing with rates from other regions, Greece has similar rates with those observed in Spain (Pesiou et al., 2023), but much less exposure to off-label psychotropics than the one reported in Iceland (Zoëga et al., 2009).

The detailed analysis of prescriptions and indications showed that several psychotropics were used in indications for which they did not have an approval. Quetiapine, which in Europe is authorized only in adults, was the most frequently dispensed off-label medicine. Antipsychotics (mainly risperidone), antidepressants (mainly fluoxetine) and antiepileptics were also found among the off-label psychotropics; however, there is available evidence worldwide providing some support to the concerned indications, coming from the existing differences in the pediatric authorization status of these medicines between Europe and the United States of America.

The off-label use in Greece is somehow regulated by a ministerial decree which is required for physicians to permit off-label prescribing. This decree is established for reimbursement reasons (Official Gazette 545/Β΄/01–03–2012) and considers authorization of off-label prescription in special cases if supported by international bibliographic references; yet, the approval must be done before use. Furthermore, Greek law 4,316/2014 states that any off-label use could be potentially reimbursed if included in therapeutic protocols approved by the central council. As in the rest of Europe, promotion of any off-label use by the Marketing Authorization Holders (MAHs) is forbidden in Greece, but MAHs remain responsible for reporting any side effects associated to this kind of use (Weda et al., 2017). Controlling off-label use is important for children with a mental health condition, because potentially severe adverse events are more frequent, as compared to adults (European Medicines Agency, 2004).

Worldwide, changes in modern psychiatry where the emphasis was shifted from the psychosocial to a medical model, resulted into a greater use of pharmacological interventions. The increasing knowledge and awareness on the negative impact of poor mental health in the normal development and social life of the pediatric population also contributed to motivating early management of these patients; this has led to increased diagnosis and subsequently treatment of psychiatric disorders with a childhood onset. Simultaneously, parental and social acceptability as well as the demand in using psychotropics in children and adolescents, have grown. Furthermore, the limited access in some countries to nonpharmacological therapeutic resources or inpatient psychiatric services, has led to psychotropics being increasingly considered as a solution for a more affordable and quick way to benefit the outpatient, and to also shorten hospitalization days (Harrison et al., 2012). In this context, the need for consistent regulatory information for the psychotropic use in pediatrics is of utmost importance.

The current study suggests that the off-label use of psychotropic agents in pediatric patients with mental health conditions is a frequent reality. Of note, off-label use implies the lack of regulatory guarantees of efficacy and safety, because of missing information and lack of evidence, or because the few available information has never been submitted as part of a marketing authorization application, due to small commercial interest. Thus, the scarce available data on neuropsychiatric indications in children and adolescents often has not been properly evaluated by regulators (Choonara and Conroy, 2002). Despite awareness on the lack of specific studies and on the risk of potentially relevant differences in pharmacokinetics, pharmacodynamics and safety between children and adults, off-label use is widely accepted as unavoidable. In fact, some studies suggest that off-label use in children, when done under closely monitored conditions, may not significantly increase risks (Neubert et al., 2004; Egberts et al., 2022). However, other guarantees such as the availability of formulations suitable for pediatric use are also missing, so that adult products are adapted to children’s requirements, risking in this way dosage errors that will continue until clinicians and regulators request evidence for this vulnerable population. Besides, regardless of short-term safety data, the sustained and crucial process of neurological and psychological maturation and development of children and adolescent may be modified by drug exposure, and whether this may impact normal functioning in the future should be properly evaluated (Gore et al., 2017). From a public health perspective, accepting routine clinical care of children without robust evidence represents a major risk, requiring urgent attention.

In Greece several difficulties impact the way mental healthcare is delivered to the pediatric patient population. Specific clinical guidelines for pediatric mental health are not established in the country. The problem highlighted by our study may support the need for nationwide guidance on how to handle the currently available psychotropics used to treat pediatric conditions and minimise their off-label use. Due to the financial crisis, the health system has deteriorated and there are several warnings on the mental-health effects of the COVID-19 pandemic, especially in children. Thus, there may be an opportunity to restart investing again in the facilities for mental health in the country (World Health Organization, 2022). A proposal for a revised medicines regulation framework was recently announced in Europe, where the regulators want to boost further development of medicines for unmet medical needs and protect more the vulnerable populations (European Commission). The value of data demonstrating the real exposure of a medicine in the broad population is considered significant in those areas where there is lack of data like in pediatric psychiatry, or when it is considered unethical to conduct clinical trials, whereas initiatives started already in the regulatory environment (US Food and Drug Administration, 2020; Arlett et al., 2022). Real-world evidence might be in the future an option to inform on the need of updating the labelling of off-patent medicines with important already available information and/or repurposing initiatives. (Mahendraratnam et al., 2022).

### 4.2 Limitations

Our study used population-based data from the public healthcare system of Greece which is representative of the country since the system covers almost all population (Bakirtzis et al., 2020). Invoicing databases generally allow exhaustive population coverage, are devoid from recall bias and thus guarantee representativeness of the studied population and drug access (Hennessy, 2006; Andersen, 2014; Sommet and Pariente, 2019). By using pharmacy claims data this study has the advantage to describe the actual exposure in psychotropics as they represent all patients actually retrieving the prescribed medicine. However, considering the possibility to purchase medicines without prescription in Greece, the quantitative data have to be set in the context of a progressive deployment of the tool and may have some underestimation of the actual drug use (Vassilakopoulou et al., 2017). The prices of most psychotropics are relatively low in Greece and those drugs can be directly obtained from the pharmacy with the exception of those under strict control from the state (e.g., benzodiazepines, opioids). Another limitation is that in this dataset there was insufficient information about the prescribed dose that precluded any further investigation. Finally, limitations include also the predetermined type of variables with the collection of new variables to be generally not possible, as well as the difficulties derived from managing large databases with data structures that are not designed for research (Andersen, 2014).

## Data Availability

The original contributions presented in the study are included in the article/Supplementary Material, further inquiries can be directed to the corresponding author.
