# Supplementary material for: Prevalence of use of on-label and off-label psychotropics in the Greek pediatric population
Source: Front Pharmacol. 2024 Mar 14;15:1348887. doi: 10.3389/fphar.2024.1348887 (PMC10972865; doi:10.3389/fphar.2024.1348887)

Supplementary Material

Prevalence of use of on-label and off-label psychotropics in the Greek pediatric population

Stella Pesiou, Rafel Barcelo, Georgios Papazisis, Ferran Torres*, Caridad Pontes

*** Correspondence:** Ferran Torres: Ferran.Torres@uab.cat

Figure 2. Off-label use as per the labelled age-range, analysis by age strata of pediatric subjects.

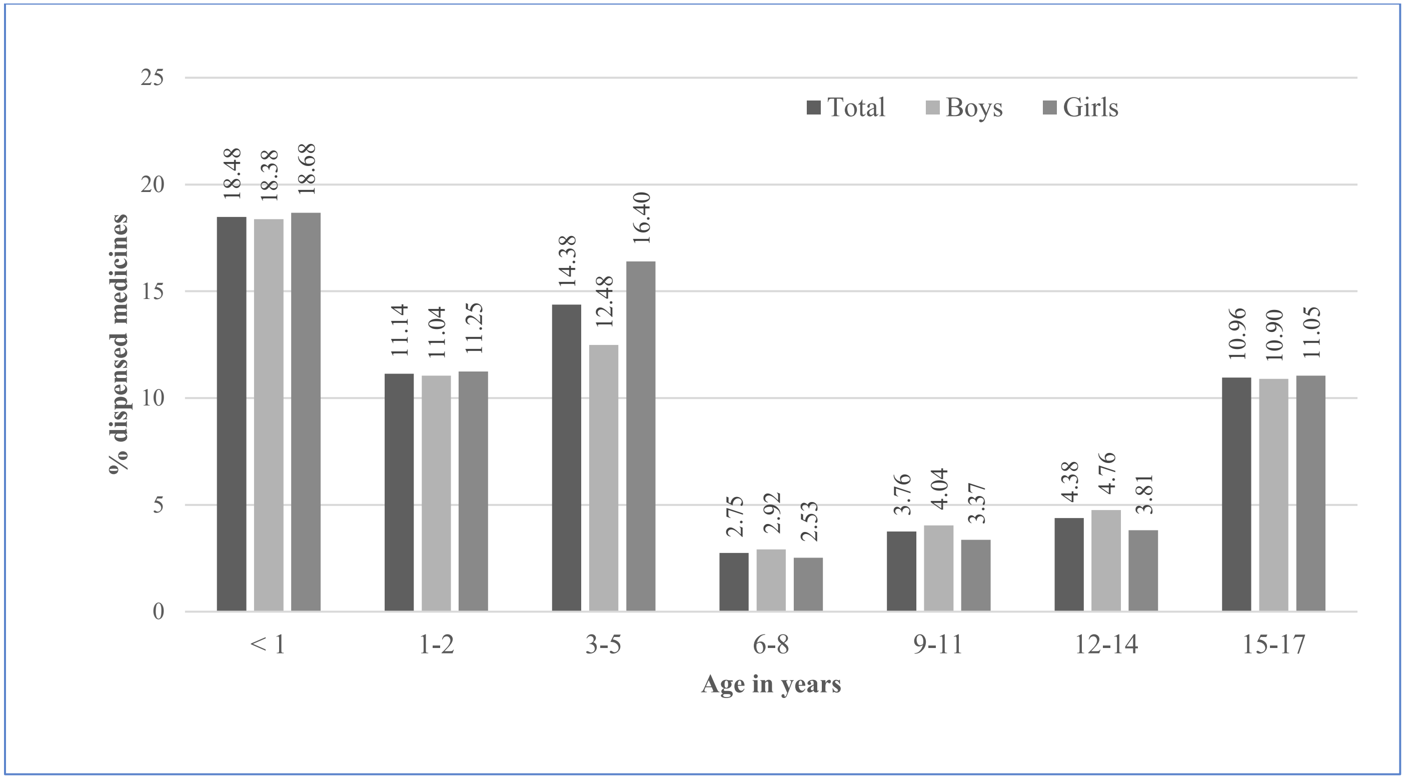

Supplement: Supplementary file 1 [file Image2.pdf]
