# Supplementary material for: Prevalence of use of on-label and off-label psychotropics in the Greek pediatric population
Source: Front Pharmacol. 2024 Mar 14;15:1348887. doi: 10.3389/fphar.2024.1348887 (PMC10972865; doi:10.3389/fphar.2024.1348887)
Supplement: Supplementary file 2 [file DataSheet1.docx]

Supplementary Material

Prevalence of use of on-label and off-label psychotropics in the Greek pediatric population

Stella Pesiou, Rafel Barcelo, Georgios Papazisis, Ferran Torres^*^, Caridad Pontes

*** Correspondence:** Ferran Torres: [Ferran.Torres@uab.cat](mailto:Ferran.Torres@uab.cat)

# Supplementary Data

Heatmap table for the most prevalent medicines used by age in 2016-2019 (Target group; N=21,269 patients).

|  |  | **Overall** | **Prev. (****‰)** | **Girls** | **Prev. (‰)** | **Boys** | **Prev. (‰)** |
| --- | --- | --- | --- | --- | --- | --- | --- |
| **< 1 year** | **1** | N05BA01: Diazepam | 1.2 | N05BA01: Diazepam | 1.1 | N05BA01: Diazepam | 1.3 |
|  | **2** | N03AA02: Phenobarbital | 1.0 | N03AX14: Levetiracetam | 1.0 | N03AA02: Phenobarbital | 1.1 |
|  | **3** | N03AX14: Levetiracetam | 0.9 | N03AA02: Phenobarbital | 0.8 | N03AX14: Levetiracetam | 0.9 |
|  | **4** | N05CD08: Midazolam | 0.4 | N05CD08: Midazolam | 0.5 | N05CD08: Midazolam | 0.3 |
|  | **5** | N03AX11: Topiramate | 0.3 | N03AX11: Topiramate | 0.3 | N03AG01: Valproic acid | 0.3 |
|  | **6** | N03AG01: Valproic acid | 0.3 | N03AG01: Valproic acid | 0.3 | N03AX11: Topiramate | 0.2 |
|  | **7** | N03AF02: Oxcarbazepine | 0.2 | N03AF02: Oxcarbazepine | 0.2 | N05BB01: Hydroxyzine | 0.1 |
|  | **8** | N05BB01: Hydroxyzine | 0.1 | N05BB01: Hydroxyzine | 0.1 | N03AF02: Oxcarbazepine | 0.1 |
|  | **9** | N03AF01: Carbamazepine | 0.1 | N03AB02: Phenytoin | 0.1 | N03AF01: Carbamazepine | 0.1 |
|  | **10** | N05BA09: Clobazam | 0.1 | N03AF01: Carbamazepine | 0.1 | N05BA09: Clobazam | 0.1 |
| **1-2 years** | **1** | N05BA01: Diazepam | 11.3 | N05BA01: Diazepam | 10.7 | N05BA01: Diazepam | 11.7 |
|  | **2** | N03AX14: Levetiracetam | 2.3 | N03AX14: Levetiracetam | 2.1 | N03AX14: Levetiracetam | 2.5 |
|  | **3** | N05CD08: Midazolam | 2.1 | N05CD08: Midazolam | 2.0 | N05CD08: Midazolam | 2.2 |
|  | **4** | N03AG01: Valproic acid | 1.4 | N03AG01: Valproic acid | 1.3 | N03AG01: Valproic acid | 1.6 |
|  | **5** | N05BB01: Hydroxyzine | 0.8 | N05BB01: Hydroxyzine | 0.8 | N05BB01: Hydroxyzine | 0.8 |
|  | **6** | N03AA02: Phenobarbital | 0.7 | N03AA02: Phenobarbital | 0.5 | N03AA02: Phenobarbital | 0.8 |
|  | **7** | N03AB02: Phenytoin | 0.4 | N03AB02: Phenytoin | 0.5 | N03AF02: Oxcarbazepine | 0.4 |
|  | **8** | N03AF02: Oxcarbazepine | 0.4 | N03AF02: Oxcarbazepine | 0.4 | N03AX11: Topiramate | 0.4 |
|  | **9** | N03AX11: Topiramate | 0.3 | N03AX11: Topiramate | 0.3 | N03AB02: Phenytoin | 0.4 |
|  | **10** | N05BA09: Clobazam | 0.3 | N05BA09: Clobazam | 0.3 | N05BA09: Clobazam | 0.3 |
| **3-5 years** | **1** | N05BA01: Diazepam | 8.0 | N05BA01: Diazepam | 7.1 | N05BA01: Diazepam | 8.9 |
|  | **2** | N03AX14: Levetiracetam | 2.3 | N03AX14: Levetiracetam | 2.2 | N03AX14: Levetiracetam | 2.4 |
|  | **3** | N03AG01: Valproic acid | 1.8 | N03AG01: Valproic acid | 1.6 | N03AG01: Valproic acid | 2.0 |
|  | **4** | N05CD08: Midazolam | 1.6 | N05CD08: Midazolam | 1.5 | N05CD08: Midazolam | 1.7 |
|  | **5** | N05BB01: Hydroxyzine | 0.7 | N05BB01: Hydroxyzine | 0.7 | N05BB01: Hydroxyzine | 0.8 |
|  | **6** | N03AF02: Oxcarbazepine | 0.7 | N03AF02: Oxcarbazepine | 0.6 | N03AF02: Oxcarbazepine | 0.7 |
|  | **7** | N05BA09: Clobazam | 0.4 | N05BA09: Clobazam | 0.4 | N05BA09: Clobazam | 0.4 |
|  | **8** | N03AX11: Topiramate | 0.3 | N03AX11: Topiramate | 0.3 | N05AX08: Risperidone | 0.3 |
|  | **9** | N03AB02: Phenytoin | 0.3 | N03AB02: Phenytoin | 0.2 | N03AB02: Phenytoin | 0.3 |
|  | **10** | N05AX08: Risperidone | 0.2 | N03AE01: Clonazepam | 0.2 | N03AX11: Topiramate | 0.3 |
| **6-8 years** | **1** | N05BA01: Diazepam | 3.1 | N03AX14: Levetiracetam | 2.9 | N05BA01: Diazepam | 3.3 |
|  | **2** | N03AX14: Levetiracetam | 2.9 | N05BA01: Diazepam | 2.8 | N03AX14: Levetiracetam | 2.9 |
|  | **3** | N03AG01: Valproic acid | 2.6 | N03AG01: Valproic acid | 2.4 | N03AG01: Valproic acid | 2.7 |
|  | **4** | N05CD08: Midazolam | 1.9 | N05CD08: Midazolam | 1.7 | N05CD08: Midazolam | 2.1 |
|  | **5** | N03AF02: Oxcarbazepine | 1.2 | N03AF02: Oxcarbazepine | 1.1 | N05AX08: Risperidone | 1.6 |
|  | **6** | N05AX08: Risperidone | 1.0 | N05AX08: Risperidone | 0.4 | N03AF02: Oxcarbazepine | 1.3 |
|  | **7** | N06BA04: Methylphenidate | 0.5 | N05BA09: Clobazam | 0.4 | N06BA04: Methylphenidate | 0.8 |
|  | **8** | N05BA09: Clobazam | 0.5 | N03AX11: Topiramate | 0.3 | N05BA09: Clobazam | 0.5 |
|  | **9** | N05BB01: Hydroxyzine | 0.4 | N05BB01: Hydroxyzine | 0.2 | N05AX12: Aripiprazole | 0.5 |
|  | **10** | N05AX12: Aripiprazole | 0.3 | N03AX09: Lamotrigine | 0.2 | N05BB01: Hydroxyzine | 0.5 |
| **9-11 years** | **1** | N03AX14: Levetiracetam | 3.5 | N03AX14: Levetiracetam | 3.5 | N03AX14: Levetiracetam | 3.6 |
|  | **2** | N03AG01: Valproic acid | 3.3 | N03AG01: Valproic acid | 2.9 | N03AG01: Valproic acid | 3.6 |
|  | **3** | N05CD08: Midazolam | 2.5 | N05CD08: Midazolam | 2.3 | N05AX08: Risperidone | 2.9 |
|  | **4** | N05BA01: Diazepam | 2.3 | N05BA01: Diazepam | 1.9 | N05CD08: Midazolam | 2.7 |
|  | **5** | N05AX08: Risperidone | 1.9 | N03AF02: Oxcarbazepine | 1.5 | N05BA01: Diazepam | 2.6 |
|  | **6** | N03AF02: Oxcarbazepine | 1.8 | N05AX08: Risperidone | 0.8 | N03AF02: Oxcarbazepine | 2.1 |
|  | **7** | N06BA04: Methylphenidate | 0.9 | N05BA09: Clobazam | 0.5 | N06BA04: Methylphenidate | 1.6 |
|  | **8** | N05BA09: Clobazam | 0.5 | N03AX09: Lamotrigine | 0.4 | N05AX12: Aripiprazole | 0.8 |
|  | **9** | N05AX12: Aripiprazole | 0.5 | N05BB01: Hydroxyzine | 0.3 | N06BA09: Atomoxetine | 0.6 |
|  | **10** | N03AX11: Topiramate | 0.4 | N03AX11: Topiramate | 0.3 | N05BA09: Clobazam | 0.6 |
| **12-14 years** | **1** | N03AX14: Levetiracetam | 3.7 | N03AX14: Levetiracetam | 3.6 | N05AX08: Risperidone | 4.0 |
|  | **2** | N03AG01: Valproic acid | 3.3 | N03AG01: Valproic acid | 2.9 | N03AX14: Levetiracetam | 3.8 |
|  | **3** | N05AX08: Risperidone | 2.9 | N05CD08: Midazolam | 2.3 | N03AG01: Valproic acid | 3.6 |
|  | **4** | N05CD08: Midazolam | 2.6 | N05AX08: Risperidone | 1.7 | N05CD08: Midazolam | 2.9 |
|  | **5** | N03AF02: Oxcarbazepine | 1.9 | N03AF02: Oxcarbazepine | 1.5 | N03AF02: Oxcarbazepine | 2.2 |
|  | **6** | N05BA01: Diazepam | 1.6 | N05BA01: Diazepam | 1.4 | N05BA01: Diazepam | 1.7 |
|  | **7** | N06AB03: Fluoxetine | 1.1 | N06AB03: Fluoxetine | 1.1 | N06BA04: Methylphenidate | 1.7 |
|  | **8** | N06BA04: Methylphenidate | 1.0 | N03AX11: Topiramate | 0.7 | N05AX12: Aripiprazole | 1.4 |
|  | **9** | N05AX12: Aripiprazole | 1.0 | N03AX09: Lamotrigine | 0.6 | N06AB03: Fluoxetine | 1.1 |
|  | **10** | N03AX11: Topiramate | 0.7 | N06AB06: Sertraline | 0.6 | N03AX11: Topiramate | 0.7 |
| **15-17 years** | **1** | N05AX08: Risperidone | 4.1 | N03AX14: Levetiracetam | 4.3 | N05AX08: Risperidone | 5.5 |
|  | **2** | N03AX14: Levetiracetam | 4.1 | N06AB03: Fluoxetine | 3.5 | N03AG01: Valproic acid | 4.3 |
|  | **3** | N03AG01: Valproic acid | 3.8 | N03AG01: Valproic acid | 3.3 | N03AX14: Levetiracetam | 3.9 |
|  | **4** | N06AB03: Fluoxetine | 2.8 | N06AB06: Sertraline | 2.9 | N06AB03: Fluoxetine | 2.0 |
|  | **5** | N06AB06: Sertraline | 2.3 | N05AX08: Risperidone | 2.6 | N05AX12: Aripiprazole | 2.0 |
|  | **6** | N05AH04: Quetiapine | 1.9 | N05BA12: Alprazolam | 2.0 | N03AF02: Oxcarbazepine | 2.0 |
|  | **7** | N05AX12: Aripiprazole | 1.7 | N05AH04: Quetiapine | 1.8 | N05AH04: Quetiapine | 1.9 |
|  | **8** | N03AF02: Oxcarbazepine | 1.7 | N05CD08: Midazolam | 1.4 | N05BA01: Diazepam | 1.7 |
|  | **9** | N05BA12: Alprazolam | 1.6 | N03AX11: Topiramate | 1.4 | N06AB06: Sertraline | 1.7 |
|  | **10** | N05BA01: Diazepam | 1.6 | N05AX12: Aripiprazole | 1.4 | N05CD08: Midazolam | 1.6 |
| **<1-17 years** | **1** | N05BA01: Diazepam | 3.5 | N05BA01: Diazepam | 3.1 | N05BA01: Diazepam | 3.8 |
|  | **2** | N03AX14: Levetiracetam | 2.4 | N03AX14: Levetiracetam | 2.4 | N03AX14: Levetiracetam | 2.4 |
|  | **3** | N03AG01: Valproic acid | 2.1 | N03AG01: Valproic acid | 1.8 | N03AG01: Valproic acid | 2.3 |
|  | **4** | N05CD08: Midazolam | 1.7 | N05CD08: Midazolam | 1.6 | N05AX08: Risperidone | 2.1 |
|  | **5** | N05AX08: Risperidone | 1.5 | N05AX08: Risperidone | 0.9 | N05CD08: Midazolam | 1.9 |
|  | **6** | N03AF02: Oxcarbazepine | 1.0 | N06AB03: Fluoxetine | 0.8 | N03AF02: Oxcarbazepine | 1.1 |
|  | **7** | N06AB03: Fluoxetine | 0.7 | N03AF02: Oxcarbazepine | 0.8 | N06BA04: Methylphenidate | 0.8 |
|  | **8** | N05AX12: Aripiprazole | 0.5 | N06AB06: Sertraline | 0.6 | N05AX12: Aripiprazole | 0.7 |
|  | **9** | N06BA04: Methylphenidate | 0.5 | N03AX11: Topiramate | 0.5 | N06AB03: Fluoxetine | 0.6 |
|  | **10** | N06AB06: Sertraline | 0.5 | N05BA12: Alprazolam | 0.4 | N05BB01: Hydroxyzine | 0.5 |

**Prevalence per ‰**
