# Supplementary material for: Prevalence of use of on-label and off-label psychotropics in the Greek pediatric population
Source: Front Pharmacol. 2024 Mar 14;15:1348887. doi: 10.3389/fphar.2024.1348887 (PMC10972865; doi:10.3389/fphar.2024.1348887)
Supplement: Supplementary file 3 [file Image1.pdf]

Supplementary Material

Prevalence of use of on-label and off-label psychotropics in the Greek pediatric population

Stella Pesiou, Rafel Barcelo, Georgios Papazisis, Ferran Torres*, Caridad Pontes

*** Correspondence:** Ferran Torres: Ferran.Torres@uab.cat

Figure 1. Prevalence (per 1,000 inhabitants) of psychotropics use by ATC groups.

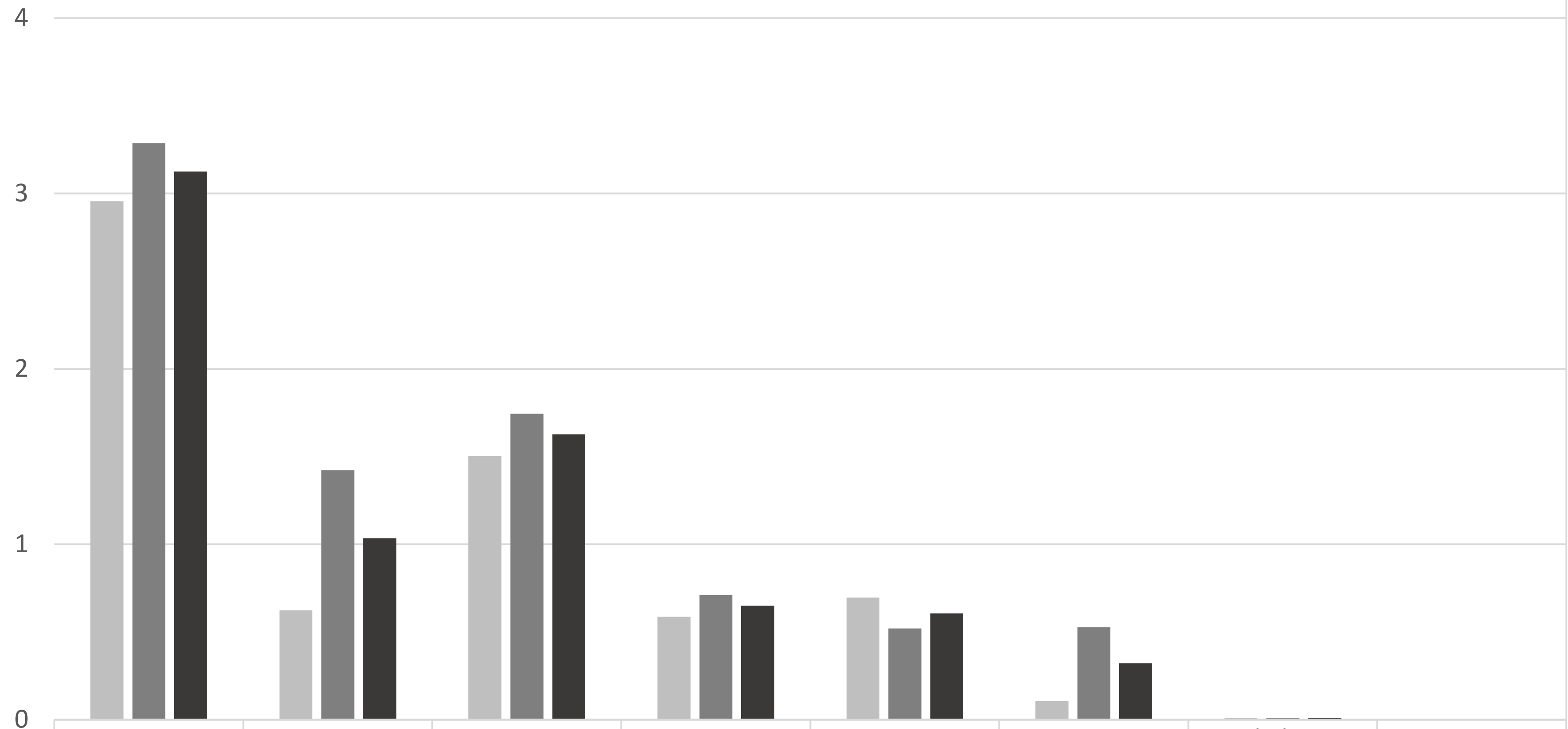

	Antiepileptics	Antipsychotics	Anxiolytics	Hypnotics and Sedatives	Antidepressants	Psychostimulants	Psycholeptics-Psychoanaleptics-Combination	Drugs - addictive disorders
Girls	2.95	0.62	1.50	0.59	0.70	0.11	0.01	0.001
Boys	3.29	1.42	1.74	0.71	0.52	0.53	0.01	0.001
Total	3.13	1.03	1.63	0.65	0.61	0.32	0.01	0.001
